# Editorial: Methods and applications in cellular neurophysiology

**DOI:** 10.3389/fncel.2023.1172741

**Published:** 2023-03-10

**Authors:** Igor Delvendahl, Bo Hu, Jonathan G. Murphy

**Affiliations:** ^1^Department of Molecular Life Sciences, University of Zurich (UZH), Zurich, Switzerland; ^2^Neuroscience Center Zurich, University of Zurich and ETH Zurich, Zurich, Switzerland; ^3^Department of Neurology, Houston Methodist Research Institute, Houston, TX, United States; ^4^Department of Developmental Neurobiology, St. Jude Children's Research Hospital, Memphis, TN, United States

**Keywords:** methods, cellular neurophysiology, electrophysiology, imaging, neural circuits, protein complexes, tissue clearing, DREADDs

## Introduction

Cellular neurophysiology is the study of the electrical and chemical activity of neurons, the basic structural and operational units of the nervous system. The history of this field can be traced back to the nineteenth century when scientists first began measuring the electrical activity of nerve cells. Early research focused on understanding the elementary properties of neurons, such as the action potential and synaptic transmission. Subsequent advances in technology allowed for more detailed studies of neural physiology and connectivity, membrane biophysics, and neuronal structure. Today, by studying the functioning of individual neurons and the mechanisms that underlie their electrical and chemical signaling, scientists can gain a deeper understanding of the physiology of the nervous system and how it is disrupted in diverse neurological or psychiatric disorders. This Research Topic assembles contributions (three research articles, three methods, and one mini review) that highlight several contemporary experimental techniques and methods used to study fundamental questions in cellular neurophysiology.

## Articles in this collection

An understanding of the spatial distribution of motor neurons, their efferent fibers, and neuromuscular targets are essential to the study of motor coordination, motor neuron impairment, and nerve repair (Levine et al., 2012). Qi et al. combined injections of multiple retrograde tracers into mouse forelimb and hindlimb muscles with 3DISCO tissue clearing to image the entire spinal cord without sectioning. The authors thus determined the three-dimensional distribution of motor neurons innervating different branches of the brachial plexus. The data could help to better understand the structural and functional connections between motor neurons and muscle fibers and improve the diagnosis and therapy of motor neuron and peripheral nerve diseases.

Activin A serves as a neuroprotective factor and has been implicated in cognitive function (Krieglstein et al., 2011). Zheng et al. used whole-cell patch-clamp recordings from dentate gyrus granule cells in activin receptor knockout mice to study how activin regulates neuronal firing. The authors found that environmental enrichment (EE) enhances neuronal excitability through non-canonical activin receptor signaling, which led to the suppression of a standing G protein-gated inwardly rectifying K^+^ (GIRK) current. This study provides a molecular mechanism linking EE to enhanced GIRK current and increased firing, potentially explaining the beneficial effects of EE on cognitive performance and affective behavior.

Designer receptors exclusively activated by designer drugs (DREADDs) allow controlling neuronal activity with single-cell precision (Armbruster et al., 2007). Gasterstädt et al. elegantly used DREADDs to dissect the role of electrical activity in dendritic and axonal maturation. The authors observed that prolonged silencing delays dendritic and axonal development of cortical pyramidal cells, possibly *via* a reduction in calcium events. The application of DREADDs technology revealed that electrical activity is a key driver in postnatal maturation of pyramidal neurons. By extension, inhibitory G-protein signaling may counterbalance growth-promoting influences during neuronal development and thus support the formation of neuronal circuits.

Olfaction begins when odorant molecules activate the olfactory sensory neurons (OSNs) in the nasal epithelium (DeMaria and Ngai, 2010). Decoding the stimulus-elicited properties of OSNs is required to understand olfactory transduction. Zak describes an *in vivo* two-photon calcium imaging method that allows for longitudinal measurements of OSN activity. Importantly, the surgically thinned cranial window permits single-cell imaging without damaging the nasal structure. Zak demonstrates robust and stable OSN responses to odorants in anesthetized and awake animals over a 21-day interval. Implementation of this approach will address challenging questions in sensory neurobiology including the role of neuromodulation, acute injury, and regeneration of OSNs in health and diseased states.

Sleep profoundly affects brain functions and promotes the consolidation of procedural and emotional memories (Diekelmann and Born, 2010). However, studying the effect of sleep on synaptic plasticity *in vivo* remains very complicated. Particularly sophisticated efforts must be made to manipulate sleep activity *in vivo*. Besing et al. present a simple way of mimicking slow-wave oscillations (SWOs) *via* up/down-states as the surrogate of sleep activity *ex vivo*. Their results show that SWOs potentiate both excitatory and inhibitory spontaneous synaptic strength in neurons and establish an effective method to study the effects of SWOs on individual neurons *ex vivo*.

Neuronal circuits consist of diverse excitatory and inhibitory neurons. Recent work has classified previously underappreciated cell types based on unique morphological, electrophysiological, and transcriptional signatures (Zeng and Sanes, 2017). Hanson and Wester review recent methods to target and manipulate defined neuron types *in vivo* to dissect their role in native neural circuits. They describe the development and use of transgenic mice and/or AAVs for cell type targeting, recently identified genetic enhancers, and intersectional fate and circuit mapping tools. The utility of genetically encoded voltage indicators and CRISPR-based genetic manipulations are described that allow for interrogation of circuit function with cell-type specificity. This timely review highlights powerful new genetic tools that can help us better understand how cortical microcircuits develop and function in health and disease.

Protein complexes are a cornerstone of cell biological processes (Marsh and Teichmann, 2015). Identifying the molecular constituents of protein complexes has important implications for understanding the physiological function of neurons. Hu et al. established a new method for identifying protein complexes and post-translational modifications in cultured hippocampal neurons. They combine lentiviral protein expression with tandem affinity purification followed by mass spectrometry to investigate neuronal K_V_4.2 potassium channel complexes. This approach offers a new way to identify protein-protein interactions and explain neuronal signaling mechanisms that may be involved in the pathophysiology of neurological diseases.

## Concluding remarks

**Figure 1:**
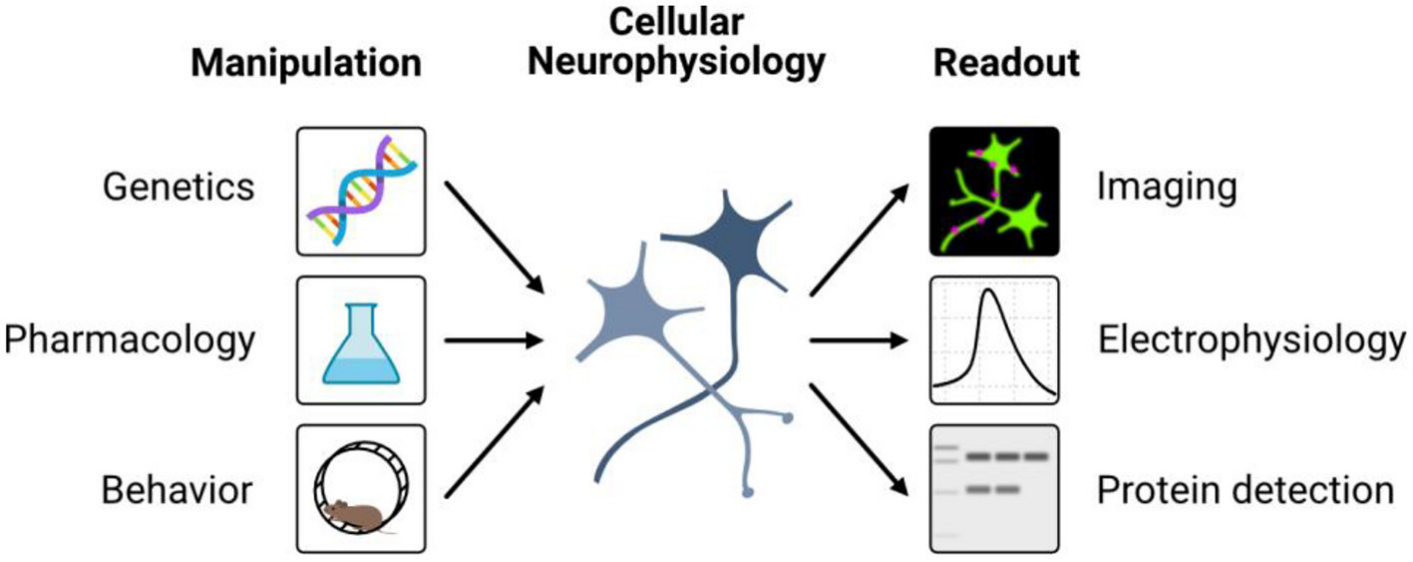
Overview of cellular neurophysiology methods reported in the articles of this collection.

The submissions to this Research Topic covered a wide range of neurophysiology methods and applications, including imaging techniques, electrophysiology, and genetic manipulations (Figure 1). Continued technological advancement will allow for more detailed and precise studies of cellular neurophysiology. We anticipate that further developments in, e.g., high-throughput techniques (Dai and Shen, 2022), high-resolution imaging approaches (Prakash et al., 2022), or machine learning (Yang and Wang, 2020) can greatly enhance the study of neural physiology. Novel methods and applications in cellular neurophysiology will thus improve our understanding of the normal functioning of the nervous system and the complex mechanisms underlying neurological disorders, leading to new and more effective treatments for these conditions.

## Author contributions

All authors listed have made a substantial, direct, and intellectual contribution to the work and approved it for publication.
